# Automatic analyser/computer system for adaptive control of phosphate concentration during fermentation

**DOI:** 10.1155/S1463924685000049

**Published:** 1985

**Authors:** M. Pécs, L. Szigeti, Gy. Lovrecz, E. Pungor, Jr., L. Nyeste, F. Holló

**Affiliations:** University of Technical Sciences Budapest Department of Agricultural Chemical Technology Gellért tér 4. Budapest H-1521 Hungary


Journal of Automatic Chemistry, Volume 7, Number 1 (January–March 1985), pages 8–19

### Automatic analyser/computer system for adaptive control of phosphate concentration during fermentation

**M. Pécs, L. Szigeti, Gy. Lovrecz, E. Pungor Jr., L. Nyeste and F. Holló**

*University of Technical Sciences, Budapest, Department of Agricultural Chemical Technology, H-1521 Budapest, Gellért tér 4., Hungary*

### Introduction

The growing demand for economical methods for optimally controlling fermentation means that more information is needed about the process. The scarcity of information about various chemical and biochemical activities during fermentation is currently one of the limiting factors to efficient control. Indirect methods, such as measurement of the rate of carbon dioxide production or oxygen uptake, have been suggested [1, 2 and 3] for estimating biomass, substrate and product concentration. At the present there are only few direct measuring instruments, for example the on-line coupled gas chromatograph, mass spectrometer, cytofluorograph and automatic analyser, because no simple sterilizable sensors are available. They all have limited applications, and for the measurement of low-volatility components only the automatic analyser (AA) seems appropriate.

Sampling is a special problem when monitoring fermentation: the solid substrates and mycelial organisms often used in industrial broths make it difficult. Continuous dialysis has successfully been used to remove solids while measuring small molecules [4 and 5], but dialysis cannot be used for large molecules (enzymes for example).

The authors have developed an automatic analyser based on continuous dialysis for general application; it has been tested for inorganic phosphate concentration control during fermentation. The present paper reports on how the system can be applied during aerobic cultivation of *Saccharomyces cerevisiae.* (The section on control theory is lengthy because the authors believe that this is the first fermentation carried out by the described method.)

### Automatic analyser applications for fermentation broth analysis

Automatic analysers are most often used for serial analysis involving hundreds of samples per day; they are useful tools in clinical, soil and environmental protection analyses. Some reports have been written about the use of AA for off-line analysis of fermentation broth [6, 7 and 8]; they have rarely been used as process-control sensors [9], probably because of the considerable delay between sampling and peak evaluation (3–30 min). So AAs can be employed only for the control of slow processes where concentration changes slowly; fermentation processes are slow (most of the variables have longer response times than AAs).

Process control needs on-line peak evaluation (peak height or peak area in the form of an electric signal). This requires at least a microprocessor-based evaluation and control system, or a computer.

The authors' AA has a sampler system which is different to that of the classic AA. The fermentor-coupled AA requires a closed sampler system to maintain sterility; this is usually fitted to a magnetic valve.

There are three possible methods for the separation of fermentation broth solids and cells: continuous dialysis [4, 5 and 8], settling [9, 10, 11, 12 and 13] and continuous filtration [13, 14 and 15] – all of these have proved to be efficient in various applications.

### Methods and equipment

### *Methods*

#### *Microorganism, cultivation conditions*

*Saccharomyces cerevisiae* yeast strain was used for test fermentations (Baker's Yeast and Alcohol Factory, Budafok, Budapest, Hungary). The baker's yeast was shaken overnight in a liquid medium on a giratory shaker at 32 °C. For shaking, phosphate-deficient medium was used containing (in grams/litre): glucose 30, $(NH_4)_2SO_4$ 5, NaCl 0·1, $MgSO_4$ 0·1, $CaCl_2$ 0·1, $KH_2PO_4$ 0·05 and biotin 0·001. 500 ml of inoculum was added into the fermentor containing 2 litres of medium (with the same composition but without phosphate). During cultivation, the agitation speed was 1000 rpm, aeration was 210 l/h (1·5 vvm), and the temperature was 30 °C.

#### *Manual method for inorganic phosphate measurement*

During the fermentation experiments, a manual phosphate analysis was run at the same time as the automatic assay. The COMECON Standards for Water and Wastewater Analytics were used [16], these are based on ammonium molybdate reaction.

#### *Gell concentration measurement*

Microbial growth was monitored by measuring optical density after a 20-fold dilution, at 620 nm, in a 1 cm cuvette. The instrument used was a SPEKTROMOM 410 spectrometer (Hungarian Optical Works, Budapest, Hungary).

### *Equipment*

### *Fermentor*

A 3 l BIOTEC laboratory fermentor body was used, coupled to a programmable control unit developed in the authors' laboratory [17].

### *Sampler unit*

AAs usually have off-line samplers – samples taken from the analysed material are manually placed in the sampler cups. A new on-line sampler unit was developed with the following characteristics:

(1) On-line (continuous) sampling directly from the fermentor.
(2) Closed system to retain sterility.
(3) Computer connection (to accept start and stop signals).

The construction of the sampler unit is shown in figure 1. Two of the magnetic valves are used by the sampler, the third has the function of control. With the decrease of response time, sample and blank solutions (wash) are circulated permanently in flexible tubing loops. Two channels of a 28-channel peristaltic pump of the AA were used for circulation. Valves 1 and 2 work inversely. As the first two rows of table 1 show, sample plugs are separated by blank solution. A detailed circuit diagram of a valve switch is shown in figure 2. Valves can be operated either by the computer or manually.

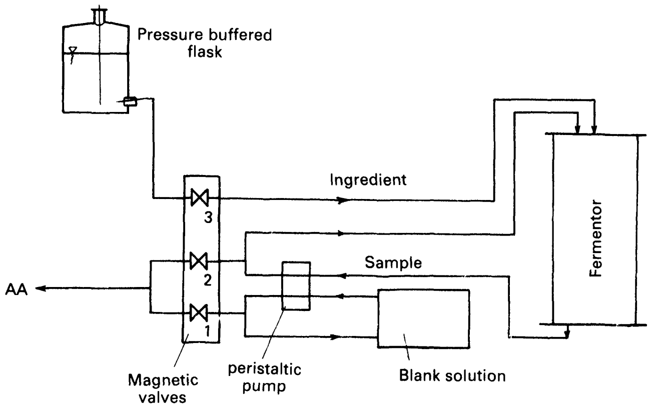

*Figure 1. Sampler and ingredient addition system.*

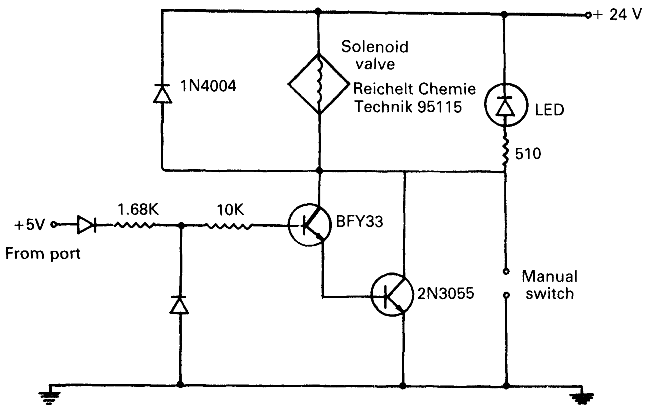

*Figure 2. Circuit diagram of valve switch.*

### *Phosphate addition*

A solution of concentrated $KH_2PO_4$ (1000 mg/l) was let into the fermentor through valve 3 from a pressure-buffered flask (see figure 1). Flow rate was always measured before the start of experiments (1·20–1·30 ml/s).

*Table 1. Time sharing of measuring and control cycle (+ means yes and – means no).*

| K | Sampling | Washing | Integration | Control | Calculation (estimation, base-line etc.) |
|---|---|---|---|---|---|
| 1 | + | – | + | – | – |
| 2 | – | + | + | + | – |
| 3 | – | + | + | + or – | – |
| 4 | – | + | – | – | + |
| 5 | – | + | + | – | – |

### *Automatic analyser*

Units of a CONTIFLO automatic analyser system (LABOR MIM, Esztergom, Hungary) were used. A reactor module for measurement of the inorganic phosphate content of blood sera (typ.: OL-704) was used with a continuous dialysator (length 184 mm). The reaction concept was as follows: under acidic conditions phosphate ions give phosphomolybdate with ammonium molybdate. This product is reduced by $SnCl_2$ to molybdene blue and the complex is measured by the two-channel photometer (typ.: OL-603) at 660 nm. The analogue output of the photometer (0–1 V) was monitored by a potentiometric recorder and put to the analogue/digital (A/D) unit. Reagent flows and segmenting air bubbles were moved by a 28-channel peristaltic pump (typ.: OL-602). The flow diagram of the system is shown in figure 3. A 1% $H_2SO_4$ solution was used as blank and reference solution.

### *Computer, interfaces*

A TRS 80 microcomputer was used in experiments (16 K ROM and 48 K RAM) and results were recorded with a line printer; other equipment included a floppy disk (5¼ in), a cassette-recorder, a tape puncher and a tape reader. Programs were written in BASIC and the interpreter was in ROM.

Input of the photometer's signal and output of valve-switching commands were organized through a programmable computer interface (INTEL 8255).

Analogue signals were converted with an A/D converter of 12 bits working in ±10 V range. The A/D was connected to a port (addresses 132 and 133: see figure 4); all bits of port 132 and the four higher bits of port 133 were input, and all bits of port 136 were output. Conversion was started by a conversion start signal

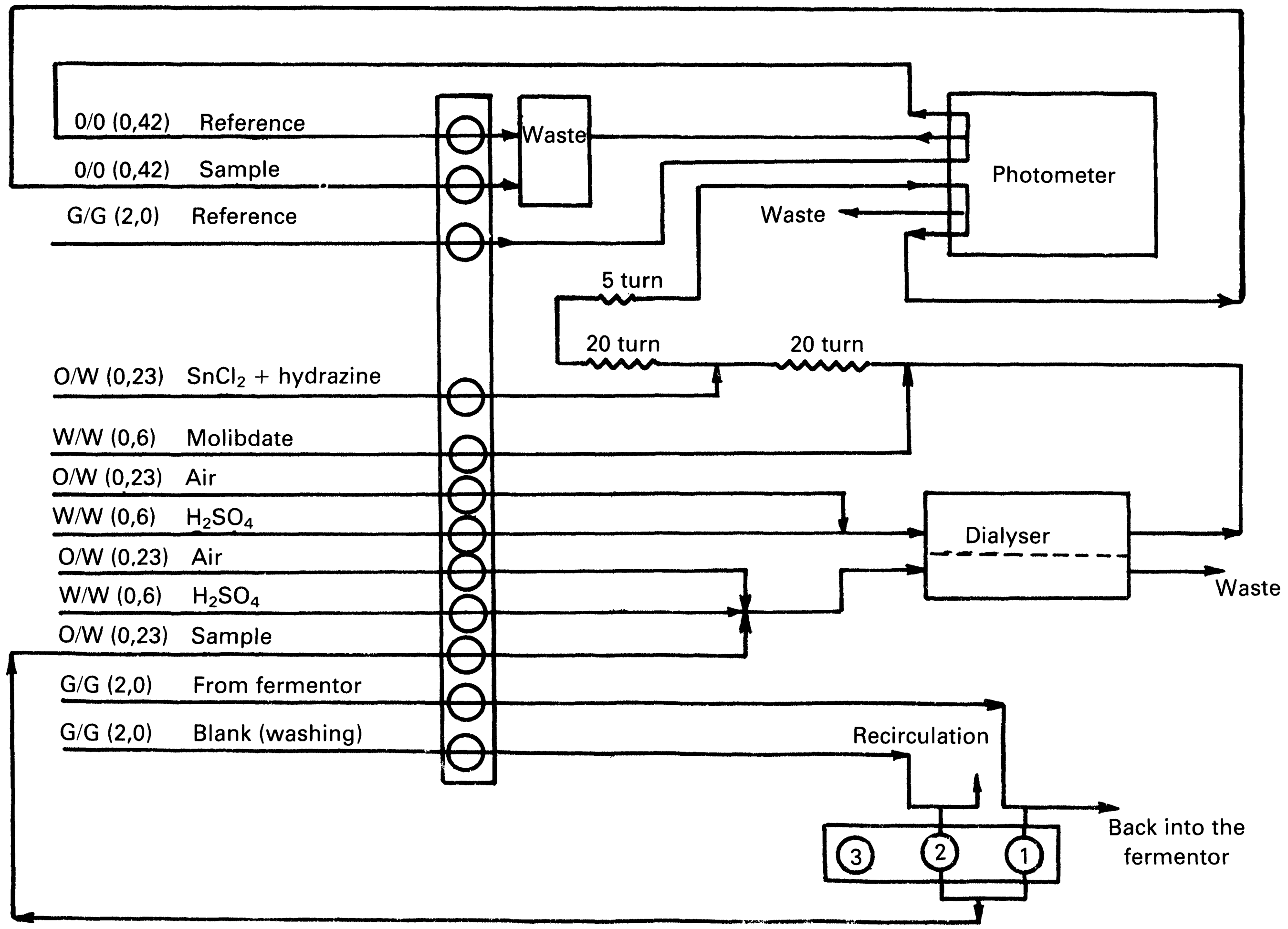

*Figure 3. Flow diagram of the analyser system.*

through port 136. Data were computed after the following commands:

OUT 136, 1 (conversion start)
X = INP (132)
Y = INP (133)
Z = 16.X + INT (Y/16)

Output commands were sent to magnetic valves via port 130 (see figure 5). The lower four bits of port 130 were output (latched), these gave out the +5 V levels for switching circuits. The higher four bits of port were input and indicated the present condition of the lower bits. Combinations determined by the codes given to the port are shown in table 2.

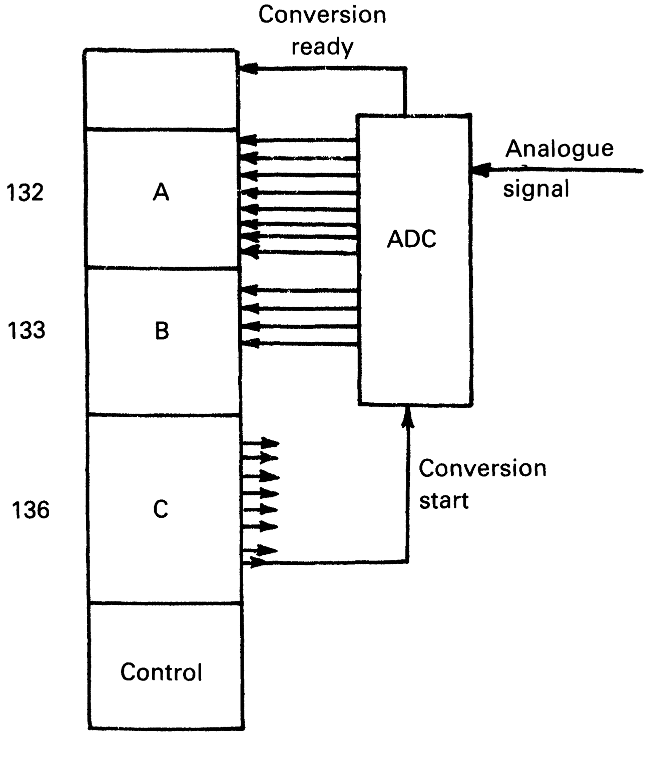

*Figure 4. Analogue/digital terminal.*

*Theoretical methods of control*

For controlling fermentation any methods can be considered which take into account random noises from the heterogeneous population and the fluctuation of external environmental conditions. These are methods of stochastic control and appear to be more suitable than methods of deterministic control.

If the aim of stochastic control is setpoint control (regulation) of one of the process variables, then the 'measure of efficiency' of control is the variance around

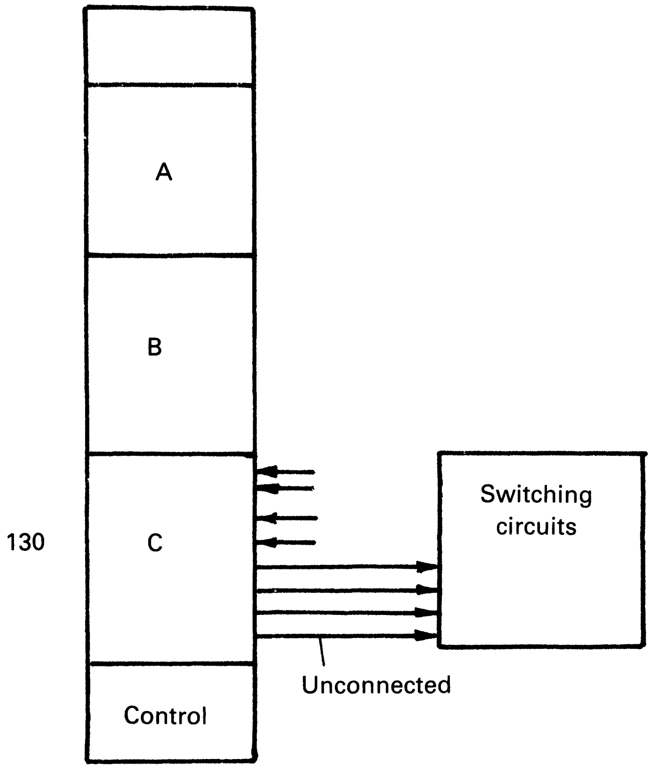

*Figure 5. Valve switch terminal.*

the setpoint. Control improves with lower variance values. The controller works well if the setpoint is controlled with minimum variance – so these controllers are known as MV (minimum variance) controllers.

On the basis of Åström's [18] proposals, linear models are used in MV controllers:

$$y(t) = \frac{B(z)}{A(z)} u(t-d) + \frac{C(z)}{D(z)} e(t). \qquad (1)$$

Where $A(z)$, $B(z)$, $C(z)$ and $D(z)$ are the polynoms of the $z^{-1}$ 'shift' operator $z^{-1}\, x(t) = x(t-1)$; $u(t)$ is the input signal, $y(t)$ is the output signal of the controller and $d$ is the deadtime.

Using the polynom separation equation [18, 19 and 20]:

$$C(z) = A(z)F(z) + Z^{-d}G(z) \qquad (2)$$

*Table 2. Effect of codes on magnetic valves.*

| | Valves 1 | 2 | 3 |
|---|---|---|---|
| 0 | Open | Open | Open |
| 1 | Open | Open | Closed |
| 2 | Closed | Open | Open |
| 3 | Closed | Open | Closed |
| 4 | Open | Closed | Open |
| 5 | Open | Closed | Closed |
| 6 | Closed | Closed | Open |
| 7 | Closed | Closed | Closed |

the value of $y$ at the $t + d$ moment can be predicted on the basis of the data up to time $t$:

$$y(t+d|t) = \frac{B(z)D(z)F(z)}{A(z)C(z)} u(t) + \frac{G(z)}{C(z)} y(t). \qquad (3)$$

If it is assumed that $A(z) = D(z)$, then the equation (3) becomes simpler:

$$y(t+d|t) = \frac{B(z)F(z)}{C(z)} u(t) + \frac{G(z)}{C(z)} y(t). \qquad (4)$$

It can be proved [18] that the control is minimum variance if:

$$y(t+d) = y_r$$

where $y_r$ is the setpoint value which is to be controlled. From equations (3) and (4) the following control law can be derived:

$$u(t) = \frac{A(z)C(z)y_r - A(z)G(z)y(t)}{B(z)F(z)D(z)} \qquad (5)$$

Then taking into account the condition $A(z) = D(z)$:

$$u(t) = \frac{C(z)y_r - G(z)y(t)}{B(z)F(z)} \qquad (5')$$

A block diagram describing the MV controller, and which is based on equation (5), is shown in figure 6. Optimal control – in the MV sense – can be given on the basis of equation (5) or (5′), if the coefficients of the polynomals, i.e. the parameters of the controllers, are known.

But in the case of a fermentation system it is practical if the parameters of controllers are not fixed, but adapted to the biological process, and the recalculation of the parameters of controllers is performed after each measurement as shown in figure 7. These are known as adaptive controllers.

For a simple description of the adaptation the $d$-step prediction can be given in linear form (19):

$$y(t+d|t) = \hat{Q}(z)u(t) + \hat{P}(z)y(t) = \boldsymbol{f}^{T}(t)\hat{\boldsymbol{p}} \qquad (6)$$

where

$$\hat{Q}(z) = q_0 + \hat{q}_1 z^{-1} + \ldots + \hat{q}_{n_q} z^{-n_q}$$

$$\hat{P}(z) = \hat{p}_0 + \hat{p}_1 z^{-1} + \ldots + \hat{p}_{n_p} z^{-n_p}$$

$$\boldsymbol{f}(t) = [u(t), \ldots u(t-n_q), y(t), \ldots y(t-n_p)]^{T}$$

$$\hat{\boldsymbol{p}} = [\hat{q}_0 \ldots \hat{q}_{n_q}, \hat{p}_0 \ldots \hat{p}_{n_p}]^{T}$$

$$n_q = m + d - 1$$

$$n_p = m + n + d - 1$$

$m$ and $n$ are the degree of polynoms $B(z)$ and $A(z)$, $T$ denotes transponation and ^ is the notation of estimated values. With this notation optimal control – in the MV sense – takes the form:

$$u(t) = \frac{y_r - \hat{P}(z)y(t)}{\hat{Q}(z)} = \frac{y_r - \boldsymbol{f}^{T}\hat{\boldsymbol{p}}(t)}{\hat{q}_0} \qquad (7)$$

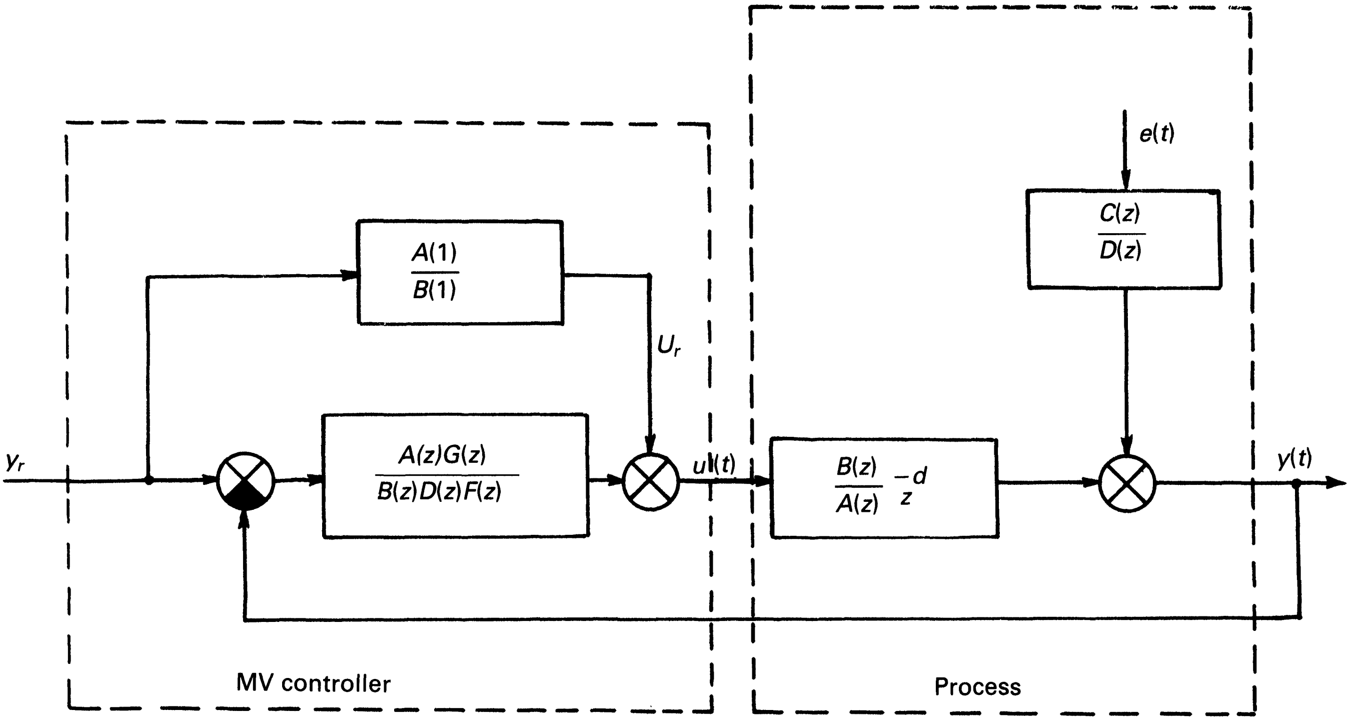

*Figure 6. Block diagram of MV controller.*

where

$$f(t) = [u(t), f(t)^T]^T$$
$$\hat{p}(t) = [\hat{q}_0, p^T]^T.$$

The predictor used in equation (6) presumes the system equation:

$$y(t+d) = f^T(t)p + \varepsilon(t+d). \quad (8)$$

(Compare equation [8] to equation [1].)

If equation (8) is described for the time moments $0 \ldots t$, the following expression is obtained:

$$\mathbf{y}_t = \mathbf{F}_{t-d}\mathbf{p} + \boldsymbol{\varepsilon}_t \quad (9)$$

where

$$\mathbf{y}_t = [y(0) \ldots y(t)]$$
$$\boldsymbol{\varepsilon}_t = [\varepsilon(0) \ldots \varepsilon(t)]$$

$$\mathbf{F}_{t-d} = \begin{bmatrix} f^T(-d) \\ f^T(1-d) \\ \vdots \\ f^T(t-d) \end{bmatrix}.$$

The following parameters:

$$\hat{p}_t = [\mathbf{F}^T_{t-d}\mathbf{F}_{t-d}]^{-1}\mathbf{F}_{t-d}\mathbf{y}_t$$

can be estimated on the basis of the first $t + 1$ $(0 \ldots t)$ measurement result by the least squares method.

The least squares method can also be realized in a recursive way, by equations (10) and (11):

$$\hat{p}_t = \hat{p}_{t-1} + \mathbf{R}_t f(t-d)[y(t) - f(t-d)\hat{p}] \quad (10)$$

$$\mathbf{R}_{t-1} = \mathbf{R}_{t-1} \frac{\mathbf{R}_{t-1} f(t-d) f^T(t-d) \mathbf{R}_{t-1}}{1 - f^T(t-d)\mathbf{R}_{t-1} f(t-d)} \quad (11)$$

so the adaptability of the controllers can be guaranteed.

Because of the closed-loop, $q_o$ has to be fixed and so equation (10) can be rewritten as:

$$\hat{p} = \hat{p}_{t-1} + \mathbf{R}_t \tilde{f}(t-d)\,[y(t) - \hat{q}_0 u(t-d) - \tilde{f}^T(t-d)\tilde{p}_t - 1]. \quad (12)$$

The control is given by:

$$u(t) = \frac{y_r - \tilde{f}^T(t)\tilde{p}_t}{\hat{q}_0}. \quad (13)$$

Fortunately it was unnecessary to fix the controller parameters for this application, thus the controller became adaptive. The number of past values concerning both input and variables, however, must be fixed.

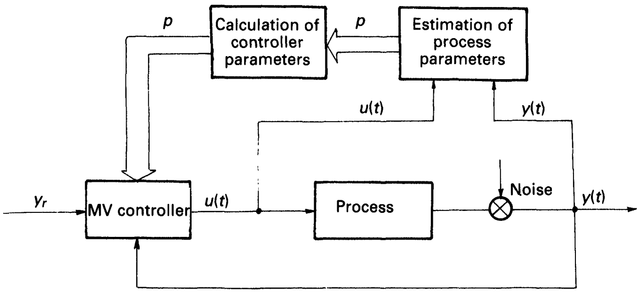

*Figure 7. The adaptive MV controller.*

(Naturally, the authors adopted the model given by equations [1] or [6].)

*The choice of the number of past values*

The correct choice of the real number of past values can be made only on the basis of experience, the following considerations, however, were helpful in making the choice.

The substrate-consuming equation [21], which is:

$$\frac{dS}{dt} = D(S_0 - S) - \frac{1}{Y}\frac{dx}{dt} \qquad (14)$$

where $D$ = dilution rate; $S_0$ = input substrate concentration; $S$ = actual substrate concentration; $Y$ = yield constant; $x$ = cell concentration.

In the simplest case it can be assumed that the change in the rate of cell concentration is proportional to cell concentration:

$$\frac{dx}{dt} = \mu x \qquad (15)$$

and between the actual substrate and cell concentration:

$$S - S^* = \frac{1}{Y}(x - x^*) \qquad (16)$$

is valid, where $S^*$, $x^*$ denotes initial substrate and cell concentrations.

Substituting $x$ from equation (16) into equation (15) and $\frac{dx}{dt}$ into equation (14), the following is obtained

$$\frac{dS}{dt} = -\mu S^* + (\mu - D)S + DS_0 + \frac{\mu}{Y}x^* =$$
$$-\mu S^* + \mu S - DS + DS_0 + \frac{\mu}{Y}x^* \qquad (17)$$

If $D\ S_0$ is considered to be a control variable (in order to realize setpoint control) and $S_0 \gg S$, the third term in equation (17) can be neglected:

$$\frac{dy}{dt} = ay + bu + c \qquad (18)$$

where $a$, $b$ and $c$ are constants.

Instead of real concentration, $y$, an integral value (peak area), is measured and the relationship between real and measured values can be modelled as a first-order lag element. This can be described in the form:

$$\frac{dy_{\mathrm{int}}}{dt} = \alpha y_{\mathrm{int}} + \beta y. \qquad (19)$$

Differentiating equation (19) with respect to $t$ and substituting equation (18), the following can be obtained:

$$\frac{d^2y_{int}}{dt^2} = \alpha\frac{dy_{int}}{dt} + \beta\,(ay + bu + c) \qquad (20)$$

then expressing $y$ from equation (19) and substituting into equation (20):

$$\frac{d^2y_{int}}{dt^2} = (\alpha + a)\frac{dy_{int}}{dt} - a\alpha y_{int} + \beta bu + \beta c\ . \qquad (21)$$

By introducing new constants, equation (21) can be given in the form:

$$\frac{d^2y_{int}}{dt^2} = a_1\frac{dy_{int}}{dt} + a_0 y_{int} + b_1 u + b_0 \qquad (22)$$

and with the aid of this equation a relationship between measured and control variables can be realized.

Using equation (22) an equation giving the number of past values as two can be obtained.

On the assumption that the controller works better if there is at least one past control variable value, the model:

$$y(t+1) = a_2 y(t) + a_1 y(t-1) + b_1 u(t) + b_2 u(t-1) + b_0 \qquad (23)$$

was chosen ($y$ is the measured peak area and $u$ is the control variable). So the coefficients of equation (23) did not agree with the coefficients of equation (22). Clearly, equation (23) can be described in the form of equation (1) by using the shift operator, and in this case $A(z)$ is a quadratic, $B(z)$ is a linear polinom, $D(z) = A(z)$ and $C(z) = 1$.

### Software

The tasks of the realized program are as follows:

(1) Determination of the integral value (peak area) from the measured data.
(2) Calculation of concentration from the integral values by a given calibration equation.
(3) Base-line correction.
(4) Control of the valves.
(5) Identifying the MV controller.
(6) Determination of the opening time of the valve which controls setpoint. This program is also suitable for simpler tasks – for example the design of experiments required for calibration equations.

The flowchart of the program is shown in figure 8. Input data are name of the experiment, sampling time, volume of fermentor, volume and phosphate concentration of ingredient tank, flow rate of the ingredient, coefficients of the calibration line, preset value of the control variable

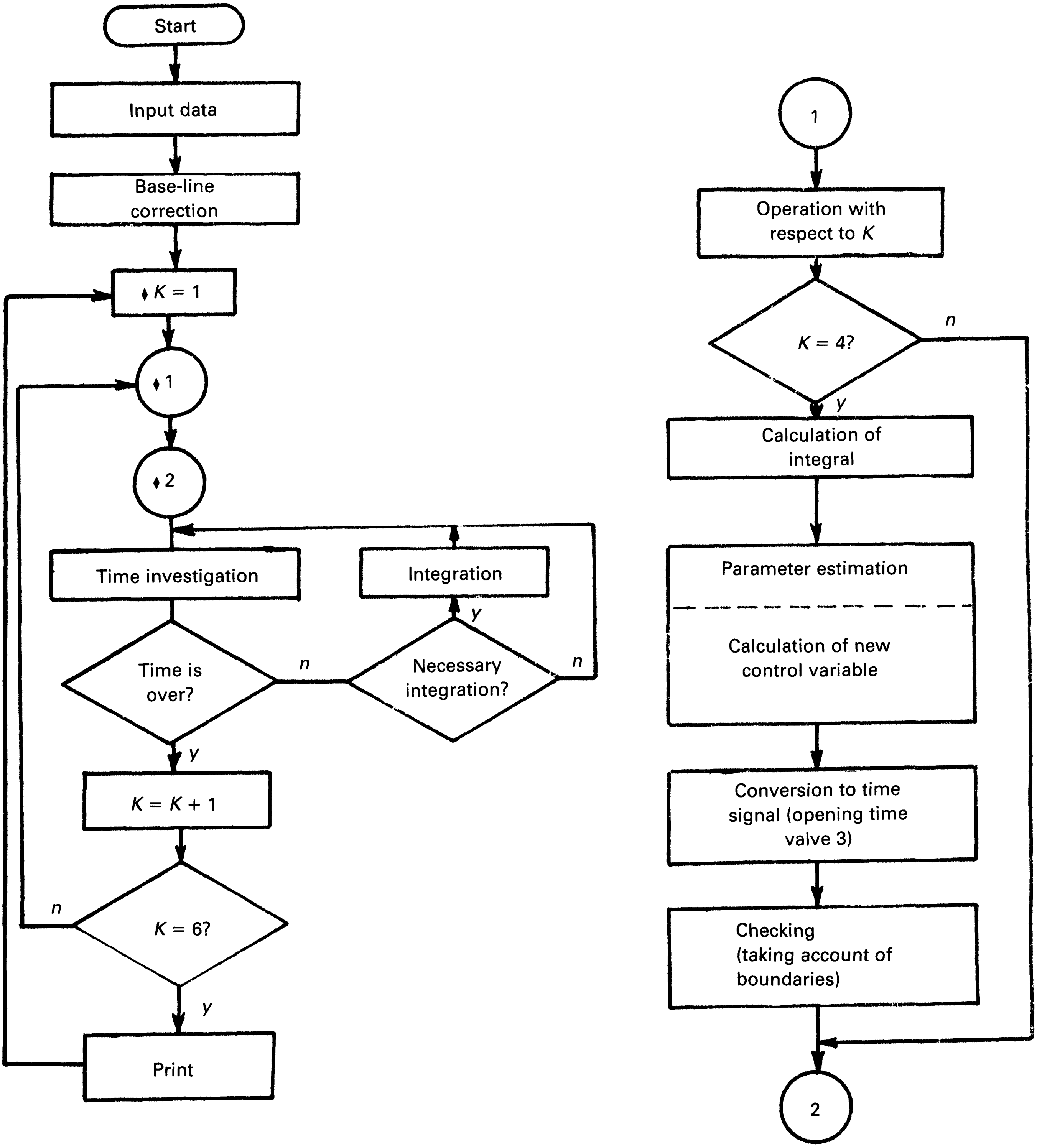

*Figure 8. The flowchart of the program.*

(setpoint), and an initial estimation of controller parameters.

The program is made clearer in table 1 and figure 9. The $K$ values are given in the flowchart (figure 8), and table 1 shows the actual state of the valve and the calculation.

The printer and the display show real time, cultivation time, phosphate concentration and phosphate addition time.

### Results and discussion

Hardware and software reported in preliminary chapters can be used for all AA measurements by changing only the reactor module. The authors decided to work with phosphate measurement for a number of reasons:

(1) It is simple and uses common reagents for both analysis and calibration.
(2) It is quite fast with a short time delay.

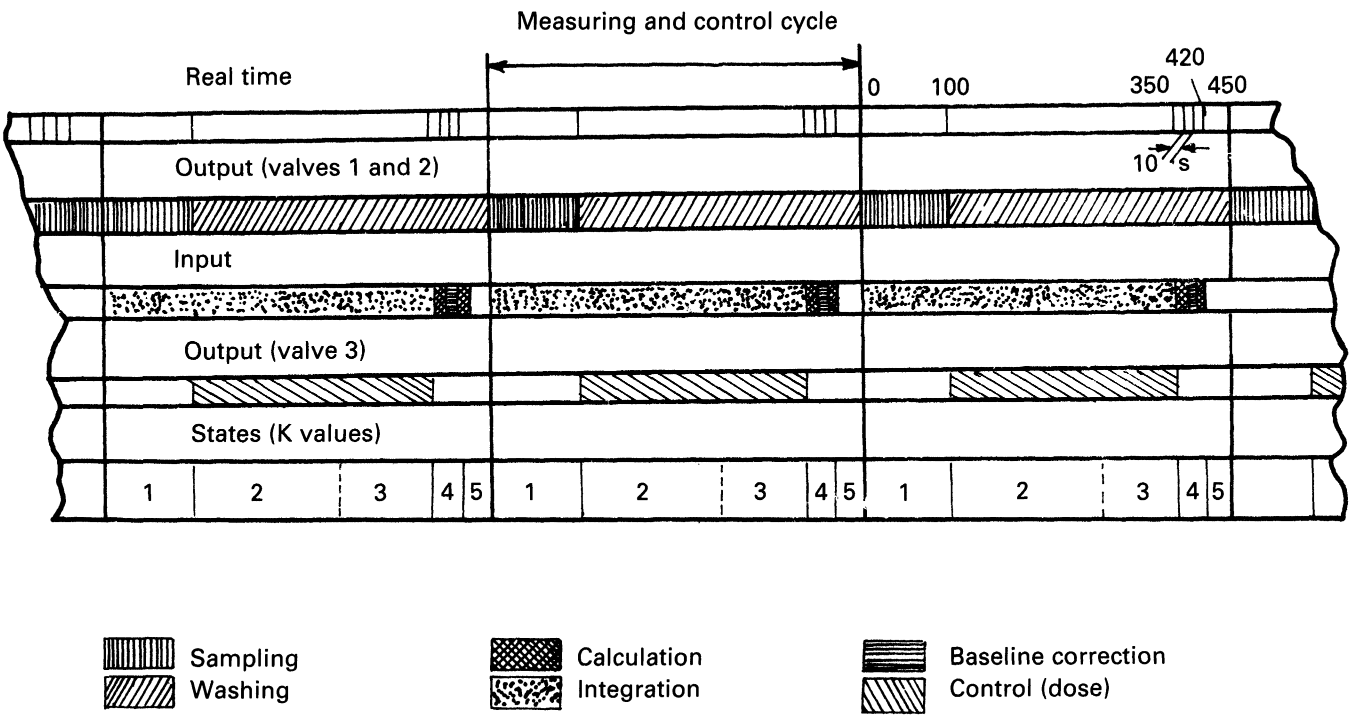

*Figure 9. Time sharing of measuring and control cycle.*

(3) Phosphate has no volatility, so it cannot be measured by any other on-line analytical devices.
(4) Phosphate has an important role in regulating fermentation processes: changes in the metabolism of microorganisms are closely connected with phosphate level [22].

*Calibration tests*

The system was initially tested with standard phosphate solutions. The software described can change sampling time during process monitoring or control. Consequently, the size of peaks depends on two factors: concentration of the analysed solution, and sampling time. Calibration was not performed on the basis of phosphate concentration but on 'phosphate amount' (PA): this was defined as: phosphate amount (mg P) = sample concentration (mg P/l) × sampling time (s) × flow rate in sample tubing (l/s). The flow rate in the tubing was constant (0·23 ml/min) so only the (concentration × time) product was used.

Another consequence of changed sampling time was deformation in the shape of the peaks. So peak heights were not proportional to phosphate amount and the areas of the peaks were used for calculations.

First, the reality of the 'phosphate amount' concept was proved. Phosphate amount values were set by different combinations of sampling times and standard phosphate concentrations (see table 3). Data belonging to the same PA values were tested by F-statistics (table 4), which led to the conclusion that there was no significant difference at 95% probability level in the studied field.

The calibration curve shown in table 4 had the following parameters:

P(0) = −2487·2 standard deviation of P(0) = 2500·1
P(1) = 32·09 standard deviation of P(1) = 0·8258
T statistics of P(0) = − 0·99
T statistics of P(1) = 38·86

written in the form of an equation:

$$\text{peak area} = 32{\cdot}09 \times \text{PA} - 2487{\cdot}2.$$

T statistics of P(0) was lower than the theoretical value of $df = 3$ and $p$ 0·95 (T = 3·187), so the intercept of the line does not differ significantly from zero.

Relative standard deviations of data groups were around 0·7 to 7%, in general they were about 4%.

*Dynamic characteristics*

The AA system had a fixed time delay. According to the programs reported there were one or two sampling cycles between sampling and result presentation (900 s). In addition to this, the magnetic valves had inner volumes of the same magnitude as sample volumes, which caused further time delays. Investigation of the response functions to unit step changes in phosphate concentration at different sampling times showed longer deadtimes at shorter sampling times (see figure 10). Therefore, shorter sampling times were not used during the experiments, in order to obtain shorter deadtimes and reliable data. This decision limited the concentration range of assay, because high concentrations were measured at very short sampling times (the top value was 1000 mg P/l at 4 s sampling).

*Table 3. Phosphate amounts (PA) and peak areas measured with different sampling times and standard phosphate solutions. All data are averages of three to six peak areas.*

| Sampling time concentration | 10 s | 20 s | 30 s | 40 s | 50 s | 60 s | 80 s | 100 s |
|---|---|---|---|---|---|---|---|---|
| | PA = 1000 | PA = 2000 | PA = 3000 | PA = 4000 | PA = 5000 | | | |
| 100 mgP/l | 27 270 | 55 991 | 94 978 | 128 689 | 152 239 | | | |
| | 27 991 | 55 080 | 100 466 | | 152 503 | | | |
| | 27 144 | 56 144 | | | | | | |
| | | PA = 1000 | | PA = 2000 | | PA = 3000 | PA = 4000 | PA = 5000 |
| 50 mgP/l | | 27 733 | | 60 035 | | 95 881 | 125 518 | 160 023 |
| | | | | | | | | 162 954 |
| | | | | | | | | 161 115 |
| | | | | | PA = 1000 | | | PA = 2000 |
| 20 mgP/l | | | | | 30 384 | | | 56 893 |

The control software took into account the sensor's time delay, so the effect of any alteration was minimalized.

*Calibration with fermentation broth*

Ten g/l of baker's yeast was suspensed in a phosphate-free medium in the fermentor. Aliquots of concentrated standard $KH_2PO_4$ solution were pipetted into the fermentor and after each addition six parallels were measured (without transients). Sampling time was 100 s, and the time of a sampling period was 450 s (see table 5). The regression line calculated was:

$$\text{P concentration (mg/l)} = 6{\cdot}33\,.\,10^{-4} \times \text{peak area} + 5.269$$

*Monitoring fermentation*

The next step in testing the hardware and software system was monitoring a real fermentation process, without control. The calibration equation estimated in the previous section was used. Baker's yeast propagation under aerobic conditions was used for testing. Sample periods were changed to 15 min and sampling time was 60 s. Cell production was measured by optical density assay at every hour of cultivation. The data recorded are presented in figure 11. There was no cell growth and no phosphate utilization during the first 2·5 h; this was the lag-period. After that, both cell growth and phosphate consumption started and ran almost parallel for the next 7 h.

*Table 4. F statistics of data groups at different PA values. There is no significant difference in any case.*

| PA (mg/l s) | Averages of data | Fisher coefficient | Degrees of freedom | Theoretical value at $P = 0.95$ |
|---|---|---|---|---|
| 1000 | 28 043 | 0·539 | 4,14 | 3·11 |
| 2000 | 56 827 | 1·84 | 4,25 | 2·76 |
| 3000 | 97 201 | 3·11 | 2,9 | 4·26 |
| 4000 | 127 501 | 1·098 | 1,8 | 5·32 |
| 5000 | 156 905 | 1·89 | 5,12 | 3·11 |

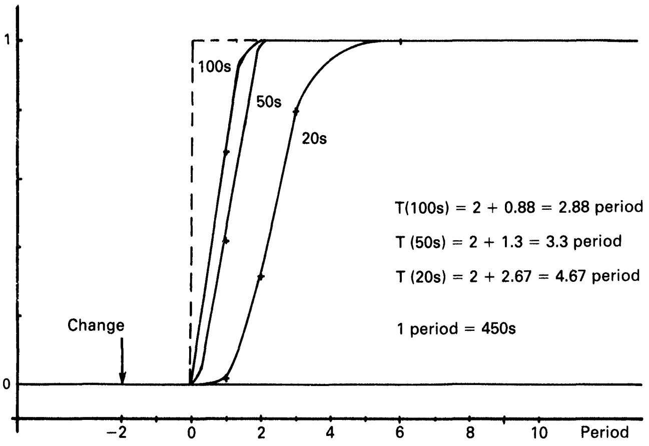

*Figure 10. Response functions of unit step changes at different sampling times.*

*Control of phosphate concentration during fermentation*

In a similar fashion to the previous experiment, baker's yeast propagation was started with 100 mg P/l initial phosphate concentration. The sample period was 450 s and the sampling time was 100 s. The preset level (setpoint) of the phosphate concentration controller was, at first, 40 mg P/l. In the initial period, when phosphate concentration was above the preset level the program worked as a monitor. When the concentration dropped below the setpoint the program added calculated amounts of phosphate solution to maintain the reference value. This part of the experiment is shown in figure 12.

*Table 5. Calibration with fermentation broth. (Sampling time = 100 s, period time = 450 s.)*

| PA (mg P/l s) | Averages of peak areas | Variances |
|---|---|---|
| 3000 | 38 398 | $1{\cdot}5306\,.\,10^6$ |
| 6000 | 85 524 | $1{\cdot}5531\,.\,10^6$ |
| 9000 | 137 409 | $1{\cdot}5186\,.\,10^8$ |
| 12 000 | 178 949 | $1{\cdot}555\,\,.\,10^7$ |

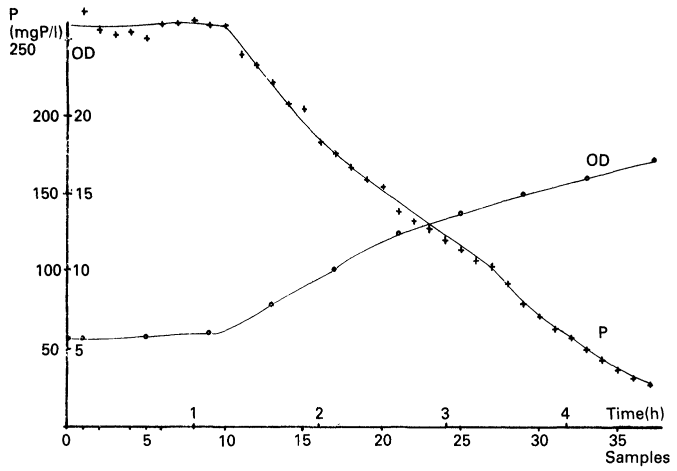

*Figure 11. Monitoring of inorganic phosphate level and optical density during aerobic cultivation of* Saccharomyces cerevisiae.

After the 4th hour of cultivation the phosphate concentration did not decrease. This was not due to a defect in the control system, but, rather, because the 30 g/l glucose, initially added, had been consumed, so both microbial growth and phosphate consumption stopped. This could be also followed in the OD curve. When this effect was realized, 100 g/l glucose was added into the fermentor, and the reference level was changed to 50 mg P/l. Microbial growth and phosphate consumption restarted and the control worked satisfactorily for 8 h.

This experiment was repeated with a higher initial glucose concentration (200 g/l), and with the addition time of phosphate solution limited (20 s inlet, 6 mg P/l concentration increase). The system worked well from both the biological and control-engineering viewpoints (see figure 13).

*Evaluation of the control experiments*

One of the fundamental problems in the study of controllers is measuring the errors made by the controller. In the theory of MV controllers, the error of the controller is equal to the measuring error.

In this case, however, the measuring error – when concentration is given by the peak area – is composed of the actual measuring error and the error of the calibration line.

The measuring error can be estimated by the variance of measurement results in the experiments. (The aim of these experiments being the determination of the calibration line.) In these cases the expression:

$$\sigma^2 = \frac{\Sigma_i^k \Sigma_j^l (y_{ij} - \bar{y}_i)^2 + \Sigma_i^k (\bar{y}_i - \hat{y}_i)^2}{kl - k + k - 2} \tag{24}$$

can be used for estimating variance [23]. Here $y_{ij}$ are the measured data; $\bar{y}_i$ are averages at a given level; $\hat{y}_i$ are estimated values of the calibration line at a given level; $k$ is the number of level of the calibration experiment; and $l$ is the number of parallels at a given level.

If some simplifying conditions are valid, then a confidence interval can be constructed (see equation [25]) around the concentration value [23]. This value can be

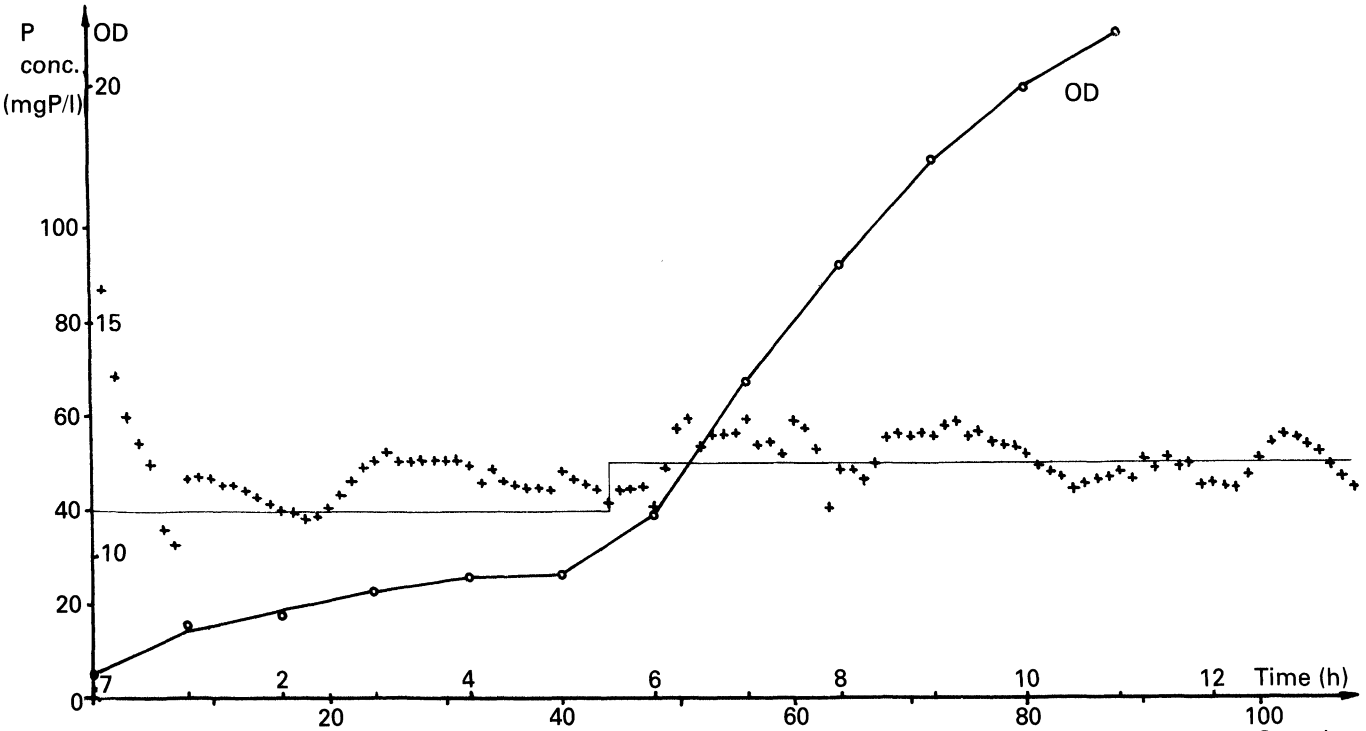

*Figure 12. Setpoint control of inorganic phosphate level by the adaptive MV controller during aerobic cultivation of* Saccharomyces cerevisiae.

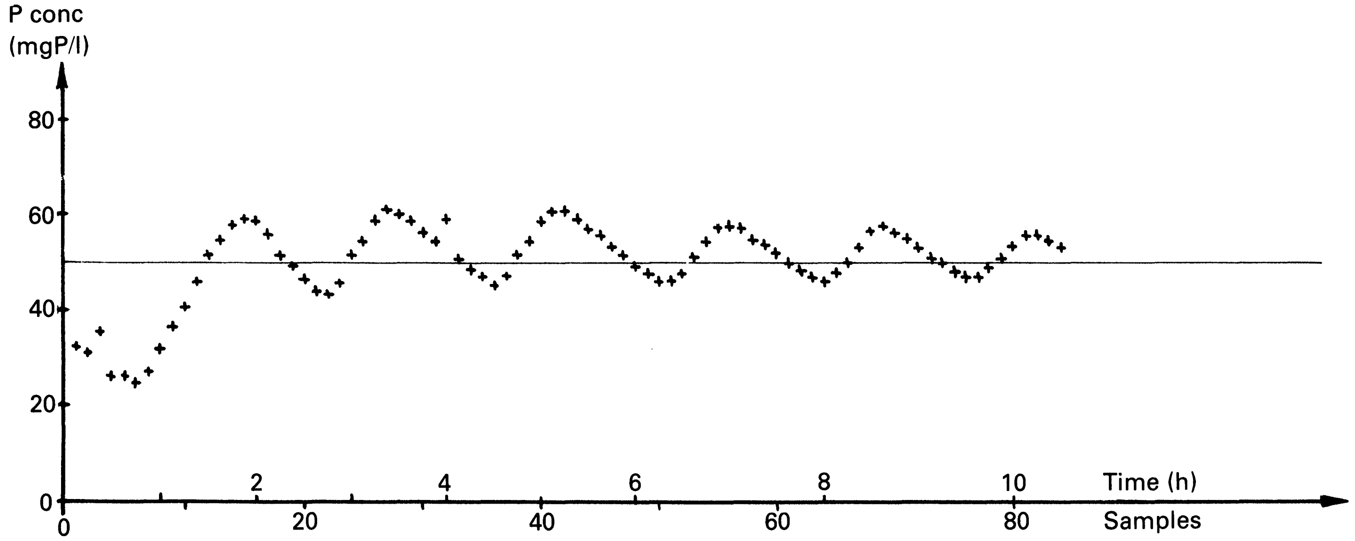

*Figure 13. Setpoint control of inorganic phosphate level by the adaptive MV controller during aerobic cultivation of* Saccharomyces cerevisiae.

calculated from the measured data and the calibration line:

$$x(y) - t_\varepsilon \frac{\sigma}{\alpha} \sqrt{1 + \frac{1}{kl} + \frac{(x(y) - \bar{x})^2}{\sum_{i=1}^{k} l(x_i - \bar{x})^2}} < x \qquad (25)$$

$$x(y) + t_\varepsilon \frac{\sigma}{\alpha} \sqrt{1 + \frac{1}{kl} + \frac{(x(y) - \bar{x})^2}{\sum_{i=1}^{k} l(x_i - \bar{x})^2}} > x$$

where $x(y)$ is the concentration belonging to the integral; $\alpha$ is the slope of the calibration line; $t_\varepsilon$ is the t (Student) value in the case of degree of freedom $k$–$l$–2 and significance level $x_i - \varepsilon$; and $x_i$ are the levels for calibration.

In this case, the expression:

$$\sigma_c = \frac{\sigma}{\alpha} \sqrt{1 + \frac{1}{kl} - \frac{(x(y) - \bar{x})^2}{\sum_{i=1}^{k} l(x_i - \bar{x})^2}}$$

can be considered as a measuring error of concentration. In the calibration experiment (see table 4), $k = 4$; $l = 6$; $x_i = 30; 60; 90; 120$; $y = 1578{\cdot}5.x - 8314$; and $\sigma = 6294{\cdot}3$. The $\sigma_c$ measuring error in the case $x(y) = 50$; $\sigma_c = 4{\cdot}0872$. The calculated controller error ($\sigma_r$) of the experiment shown in figure 12 is $\sigma_r = 6{\cdot}6177$ (66 data; 8·25 h). The setpoint value was 50.

The calculated error of the controller during the experiment presented in figure 13 was $\sigma_r = 4{\cdot}31921$ (49 data; 6·125 h). The setpoint was the same.

To compare measuring and controller errors an F-test had to be realized. In the first experiment:

$$F_{65,22} = 2{\cdot}621 > 1{\cdot}89 = F\ (0{\cdot}95),$$

and in the second experiment:

$$F_{48,22} = 1{\cdot}116 < 1{\cdot}91 = F\ (0{\cdot}95),$$

(where Fs are the actual values of table F).

Thus it can be shown that the performance of the MV controller was excellent in the second experiment – there was no significant difference between measuring and controller errors. In the first experiment the error of the controller was greater than the measuring error (this was probably due to the metabolic changes mentioned earlier).

### Conclusion

The experimental results show successful on-line measuring and control of one component in fermentation broths by a computer-coupled autoanalyser system. The most important results were:

(1) A computer-controlled magnetic valve sampler unit was developed.
(2) An autoanalyser was transformed for measuring untreated fermentation broth by continuous dialysis.
(3) Programs were developed for handling an autoanalyser as a sensor of controller.
(4) Software was developed for adaptive process control.
(5) The autoanalyser-computer-controller system was tested on baker's yeast fermentation and monitoring and control of inorganic phosphate in fermentation broth was achieved.

Optimal control of fermentation processes is no longer a question of algorithms or computers, rather, it depends on the sensors and measuring techniques applied. Computer-coupled on-line analytical devices are now required for measuring chemical substances. This study offers a potential pathway in this direction.

### Acknowledgement

The authors wish to thank the Hungarian Academy of Sciences for financial support.

### References

1. Humphrey, A. E., *Process Biochemistry*, **12** (1977), 19.
2. Cooney, C. L., Wang, H. and Wang, D. I. C., *Biotechnology and Bioengineering*, **18** (1976).
3. Carlberg, G., Holmberg, A. and Sievänen, R., *Fermentation of Bac. thüringiensis for exotoxin production – A Process analysis study.* (Helsinki University of Technology, Systems Theory Laboratory, Report B51,(1978).
4. Mor, J.-R., Zimmerly, A. and Fiechter, A., *Analytical Biochemistry*, **52** (1974), 614.
5. Zabriskie, D. W. and Humphrey, A. E., *Biotechnology and Bioengineering*, **20** (1978), 1295.
6. Roy, R. B. and Buccafuri, A., *Journal of Automatic Chemistry*, **2** (1980), 159.
7. Högye, I. and Nagy, Cs., *Proceedings 5th Fermentation in Colloquium* (Debrecen, Hungary, 1981), 184.
8. Benke, J., Automatic assay for L-tryptophan determination in fermentation broth. (B. Sc. Thesis, University of Technical Sciences, Budapest, 1981).
9. Dean, J. R. and Stewart, R. B., *Journal of Automatic Chemistry*, **1** (1979), 252.
10. Ojamo, H. and Leisola, M., *27th IUPAC Congress* (Helsinki, 1979), 575.
11. Leisola, M., Ojamo, H. and Kauppinen, V., *Enzyme Microbiological Technology*, **1** (1979), 51.
12. Leisola, M., Ojamo, H., Kauppinen, V., Linko, M. and Virkkunen, J., *Enzyme Microbiological Technology*, **2** (1980), 121.
13. Leisola, M. and Kauppinen, V., *Biotechnology and Bioengineering*, **20** (1978), 837.
14. Kuhlmann, W., Meyer, H. D. and Schügerl, K., *Biotechnologie und Verfahrenstechnik*, **16** (1982), 518.
15. Hill, F. F. and Thommel, J., *Process Biochemistry*, **17** (1980), 16.
16. Standardized assays for water investigation. Chemical methods. COMECON Standards (VITUKI, Budapest, 1968).
17. Nyeste, L., Szigeti, L., Veres, A., Pungor, E. Jr., Kurucz, I. and Holló, J., *Biotechnology and Bioengineering*, **23** (1981), 405.
18. Åström, K. I., *Introduction to Stochastic Control Theory* (Academic Press, New York, 1970).
19. Keviczky, L., New Adaptive Discrete MV controllers. D.Sc. Dissertation (Budapest, 1979).
20. Keviczky, L. and Hetthéssy, J., *Periodica Polytechnica*, **18** (1974), 291.
21. Pirt, S. J., *Principles of Microbe and Cell Cultivation* (Blackwell, Oxford, 1975).
22. Martin, J. F., Control of antibiotic synthesis by phosphate. In Ghose, T. K., Fietcher, A. and Blakebrough, N., *Advances in Biochemical Engineering* **6**, 105 (Springer Verlag, Berlin, 1977).
23. Vincze, I., *Mathematical Statistics with Industrial Applications* (Müszaki Könyvkiadó, Budapest, 1968).

### PRODUCT DESIGN ASSURANCE IN ENGINEERING

The Society of Environmental Engineers' 11–13 June 1985 conference at Wembley will cover Specifications, Techniques and Case studies.

In conjunction with the conference there will be a major exhibition of environmental equipment.

*Details from Mrs Helen Gibbons, Society of Environmental Engineers Secretariat, Owles Hall, Buntingford, Hertfordshire, UK. Tel.: 0763 71209.*

### NOTES FOR AUTHORS

*Journal of Automatic Chemistry* covers all aspects of automation and mechanization in analytical, clinical and industrial environments. The *Journal* publishes original research papers; short communications on innovations, techniques and instrumentation, or current research in progress; reports on recent commercial developments; and meeting reports, book reviews and information on forthcoming events. All research papers are refereed.

### Manuscripts

Two copies of articles should be submitted to the Editor. All articles should be typed in double spacing with ample margins, on one side of the paper only. The following items should be sent: (1) a title-page including a brief and informative title, avoiding the word 'new' and its synonyms; a full list of authors with their affiliations and full addresses; (2) an abstract of about 250 words – this should succinctly describe the scope of the contribution and highlight significant findings or innovations; (3) the main text; (4) appendices (if any); (5) references; (6) tables, each table on a separate sheet and accompanied by a caption; (7) illustrations (diagrams, drawings and photographs) numbered in a single sequence from 1 upwards and with the author's name on the back of every illustration; captions to illustrations should be typed on a separate sheet. Papers are accepted for publication on condition that they have been submitted only to *Journal of Automatic Chemistry.*

### References

References should be indicated in the text by numbers following the author's name, i.e. Skeggs [6]. In the reference section they should be arranged thus:

to a journal

Manka, D. P., *Journal of Automatic Chemistry*, **3** (1981), 119.

to a book

Malmstadt, H. V., in *Topics in Automatic Chemistry*, Ed. Stockwell, P. B. and Foreman, J. K. (Horwood, Chichester, 1978), p. 68.

### Illustrations

Original copies of diagrams and drawings should be supplied, and should be drawn to be suitable for reduction to the page or column width of the *Journal*, i.e. to 85 mm or 179 mm, with special attention to lettering size. Photographs may be sent as glossy prints or as negatives.

### Proofs and offprints

The principal or corresponding author will be sent galley proofs for checking and will receive 50 offprints free of charge. Additional offprints may be ordered on a form which accompanies the proofs.

*Manuscripts should be sent to either Dr P. B. Stockwell or Ms M. R. Stewart,* *see p. 7.*

